# Systematic Investigation of Insulin Fibrillation on a Chip

**DOI:** 10.3390/molecules25061380

**Published:** 2020-03-18

**Authors:** Hoon Suk Rho, Henk-Willem Veltkamp, Alexander Thomas Hanke, Marcel Ottens, Christian Breukers, Pamela Habibović, Han Gardeniers

**Affiliations:** 1 Department of Instructive Biomaterials Engineering, MERLN Institute for Technology-Inspired Regenerative Medicine, Maastricht University, 6200 MD Maastricht, The Netherlands; h.rho@maastrichtuniversity.nl (H.S.R.); p.habibovic@maastrichtuniversity.nl (P.H.); 2 Mesoscale Chemical Systems Group, MESA+ Institute for Nanotechnology, University of Twente, 7522 NB Enschede, The Netherlands; 3 Integrated Devices and Systems Group, MESA+ Institute for Nanotechnology, University of Twente, 7522 NB Enschede, The Netherlands; h.veltkamp@utwente.nl; 4 BioProcess Engineering Group, Department of Biotechnology, Faculty of Applied Sciences, Delft University of Technology, 2628 CD Delft, The Netherlands; athanke@gmail.com (A.T.H.); m.ottens@tudelft.nl (M.O.); 5 Medical Cell BioPhysics Group, Technical Medical Centre, University of Twente, 7522 NB Enschede, The Netherlands; c.breukers@utwente.nl

**Keywords:** microfluidics, high-throughput screening, insulin fibrillation, dual concentration gradients

## Abstract

A microfluidic protein aggregation device (microPAD) that allows the user to perform a series of protein incubations with various concentrations of two reagents is demonstrated. The microfluidic device consists of 64 incubation chambers to perform individual incubations of the protein at 64 specific conditions. Parallel processes of metering reagents, stepwise concentration gradient generation, and mixing are achieved simultaneously by pneumatic valves. Fibrillation of bovine insulin was selected to test the device. The effect of insulin and sodium chloride (NaCl) concentration on the formation of fibrillar structures was studied by observing the growth rate of partially folded protein, using the fluorescent marker Thioflavin-T. Moreover, dual gradients of different NaCl and hydrochloric acid (HCl) concentrations were formed, to investigate their interactive roles in the formation of insulin fibrils and spherulites. The chip-system provides a bird’s eye view on protein aggregation, including an overview of the factors that affect the process and their interactions. This microfluidic platform is potentially useful for rapid analysis of the fibrillation of proteins associated with many misfolding-based diseases, such as quantitative and qualitative studies on amyloid growth.

## 1. Introduction

Several common neurodegenerative disorders, such as Parkinson’s disease, type II diabetes, and Alzheimer’s disease, are known to be related to amyloidosis, in which innoxious proteins change into amyloid fibrils [1,2,3,4]. Understanding the fundamental mechanism and critical parameters in the formation of amyloid fibrils is critical for developing strategies to interrupt or reverse amyloid fibrillation and treat diseases caused by severe protein conformational misfolding [4,5]. The main parameters that affect protein fibrillation are identity, purity, and concentration of protein and environmental factors, such as pH, ionic strength, mechanical agitation, and temperature [5,6,7,8,9,10]. Moreover, transient partially folded proteins are thought to be closely related to fibril formation, e.g., by acting as fibril precursors [1,7].

Insulin is a small protein with a molecular weight of 5.7 kDa that has α-helical structures in the native state [3,11]. However, the protein converts into amyloid fibrillar structures under appropriate conditions [6]. Insulin is commonly used as a model system to evaluate the mechanism of amyloid aggregation because the structural properties of insulin fibrils are similar to those of other amyloidogenic proteins [11,12,13]. Previous in vitro studies characterized the influences of temperature, pH, agitation, and ionic strength on the aggregation of insulin through various techniques [5,12,13,14]. Batch incubation of insulin solution under different conditions is the most common technique to form insulin fibrils in laboratories [5,7,9]. Even though the traditional incubation method successfully identified the critical parameters affecting the formation of insulin fibrils and the growth of insulin fibrillar structures, multiple sample preparation steps and long incubation times are required to systematically evaluate the (intertwined) effects of a large number of factors. Besides, the conventional method for the kinetic study of protein fibrillation phenomena is limited to the observations of the early stage growth of fibrils only due to the large sample volume, which requires additional processes to analyze the number of fibrils or fibrillar structures.

To address these challenges, several microfluidic platforms have been developed to study protein-folding processes [2,15,16,17,18,19,20,21,22,23,24,25,26]. The potential of miniaturized systems for fundamental studies of protein aggregation was shown by the examples of microreactors [2,27], microchannel networks [15,19,20,21,23,24], and microdroplets [16,17,18,22,25,26]. Laminar flow in microfluidic channels enabled the characterization of protein-refolding yield [15], protein aggregates polymorphism [21], and protein aggregation phenomena [24]. Droplet-based microfluidics offered dimensional scaling benefits that enable us to reduce more of the sample volume [16,17,18,22,25,26], resulting in the detection of single primary protein nucleation and spatial propagation [22]. Moreover, microfluidic systems have been shown to have great promise as a tool for the characterization and separation of protein fibrils and aggregates by adapting single-molecule fluorescence [28], combined space and time data analysis [29], and electrophoresis [30,31]. Recent advances in microfluidic technologies, e.g., fast analysis, decreasing sample consumption, and automated flow control, enabled the increase in sensitivity and throughput. However, creating various incubation conditions by combining multiple concentration gradients of reagents remains challenging for the quantitative characterizations and kinetic studies of insulin fibrillation. Therefore, the development of a highly automated and integrated system is of great importance. Previously, microfluidic devices made by multilayer soft lithography [32,33] showed the potential of the parallelization of microreactors as a fast and automated diagnostic tool for biological and biotechnological applications, including protein crystallization [34,35], enzyme kinetics [36], DNA amplification [37,38,39,40], and cell culture [41,42]. However, the beneficial aspects of the large-scale integration of microfluidic reactors for high-throughput screening have not yet been exploited to address the challenge of the fast evaluation of protein fibrillation under various conditions with extremely small sample volumes.

Here, we developed a microfluidic protein aggregation device (microPAD) that enabled a series of protein incubations under various conditions. The device comprises 64 parallel incubation chambers to conduct 64 individual protein-folding reactions with varying concentrations of two factors. Using the microfluidic chip, we demonstrated nanoliter-scale bovine insulin aggregations, to evaluate the combined effect of concentrations of sodium chloride (NaCl) and hydrochloric acid (HCl) on the formation of insulin fibrils and fibrillar superstructures. Incubation of insulin, present in each reaction chamber in the same amount, was performed by using combinations of eight different concentrations of NaCl and HCl, followed by monitoring of insulin fibril and spherulites formation, using a fluorescent marker (Thioflavin T).

## 2. Results and Discussion

### 2.1. Design and Fabrication of a MicroPAD

**Figure 1:**
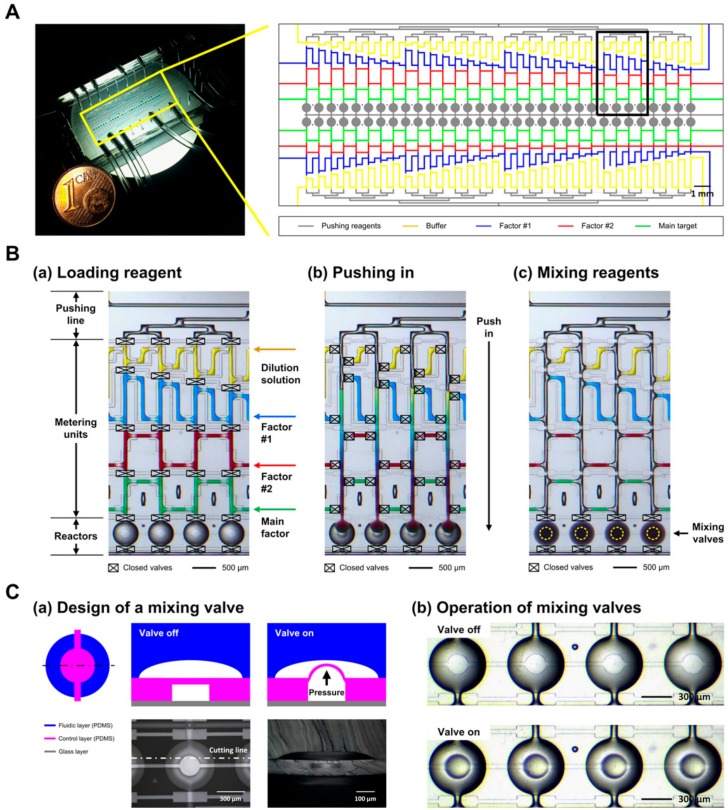
Design and operation of the microPAD. (**A**) Design of the device. (**B**) Operation of the device flows through (**a**) loading and metering, (**b**) pushing in, and (**c**) mixing. (**C**) (**a**) Design of a mixing valve and (**b**) operation of the mixing valves.

The device consists of 64 incubation units. Each unit consists of a pushing line, a metering unit, and an incubation chamber (Figure 1A,B). Figure 1B shows the step-by-step operation of the device. The operation steps include (1) loading the reagents, (2) pushing the metered reagents into reaction chambers, and (3) mixing the reagents by using mixing valves located in the center of the chamber (Movie S1 in the Supplementary Materials). The metering unit comprises four loading sites: a dilution solution site (yellow color), a factor #1 site (blue color), a factor #2 site (red color), and a main factor site (green color). The samples were loaded by pressurizing them from the inlets while the central valves were closed and the side valves in the metering units were open (Figure 1B(a)). The metering units were designed to create stepwise gradients of two reagents, i.e., in the ratios of 1:1, 1:1.57, 1:2.13, 1:2.7, 1:3.27, 1:3.83, 1:4.4, and 1:4.97, for each reagent (Table 1). After the metering of the reagents, the central valves were closed, the side valves were opened, and the reagents were pushed into the incubation chambers (Figure 1B(b)). Then, all valves were closed, and the reagents were mixed by the mixing valves (Figure 1B(c)). Figure 1C shows the design and operation of the mixing valves. The valves were designed to push up a certain volume at the center of the incubation chamber by actuation of the membrane between a fluidic channel and a control channel (Figure 1C(a)) [39,43,44]. The actuation height of the membrane is controlled by a pressure applied via the control channel, as was shown by simulation and testing (Figure S1 in the Supplementary Materials). The optimal pressure and operating frequency of the valve to mix reagents in the chamber were determined to be 0.2 bar and 1.0 Hz, respectively. Microscope images in Figure 1C(b) show de-actuation (top) and actuation (bottom) of four mixing valves. The operation of 8 mixing valves is shown in Movie S2 in the Supplementary Materials. The mixing efficiency was accessed by observing average brightness-value changes in incubation chamber areas during the mixing of the dye solutions (Figure S2 in the Supplementary Materials) [39]. The mixing of the loaded solutions in the incubation chambers was completed in less than 20 s (*n* = 8).

### 2.2. Calibration of the MicroPAD

**Figure 2:**
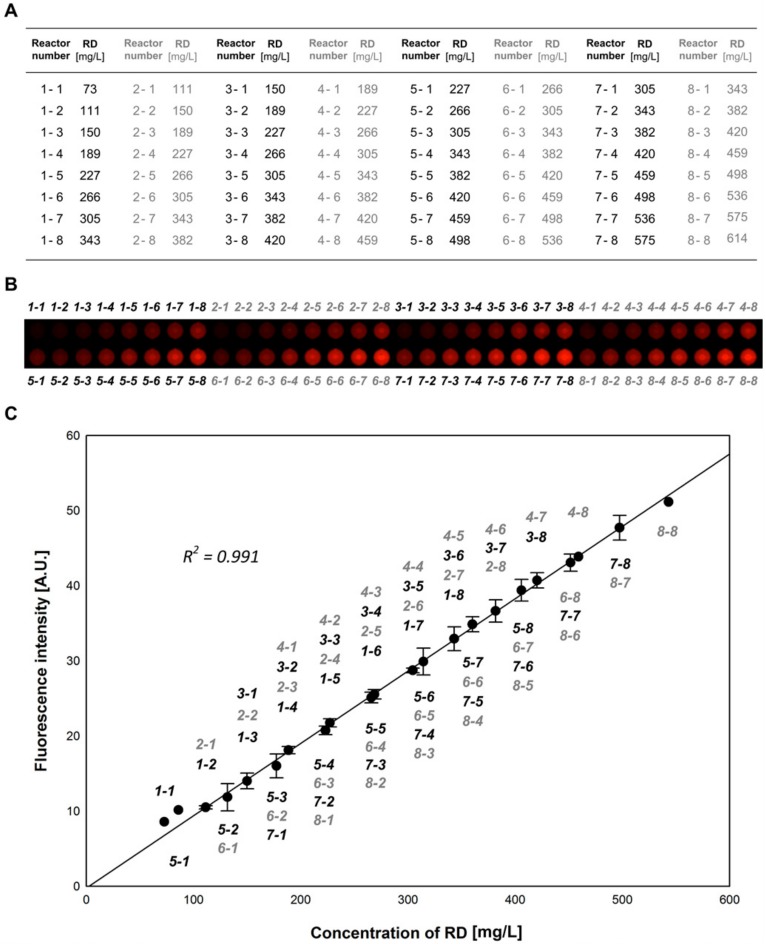
On-chip concentration gradient of RD. (**A**) Calculated final concentrations of RD in the 64 incubation chambers. (**B**) An acquired fluorescence image of the 64 parallel incubation chambers. (**C**) The relationship between the calculated concentrations of RD and the obtained RD fluorescent intensities in the chambers.

For characterization of the metering and mixing functionality of the device, concentration gradients of Rhodamine B isothiocyanate-Dextran (RD) were formed on a chip. Then, 1 g/L of RD solution was introduced into dilution solution loading sites of the metering units, while factor #1-, factor #2-, and main factor loading sites were filled with Milli-Q water. After loading, the reagents were pushed into the incubation chambers and mixed for 3 min by operating the mixing valves. The final concentration of RD ranged from 73 to 543 mg/L (Figure 2A). In Figure 2B, the fluorescence image presents the concentration gradient of RD in 64 parallel incubation chambers, and Figure 2C shows the obtained fluorescence intensities of the chambers.

### 2.3. The Effect of Insulin Concentration on Insulin Fibrillation

The effect of the insulin concentration on insulin fibrillation was studied in 64 microfluidic incubation chambers. HCl solution containing 50 mM HCl and 20 µM ThT; bovine insulin solution with 20 mg/mL bovine insulin; 50 mM HCl and 20 µM ThT HCl solution containing 50 mM HCl and 20 µM ThT; and NaCl solution containing 300 mM NaCl 50 mM HCl and 20 µM ThT were introduced into dilution solution sites, factor #1 sites, factor #2 sites, and main factor loading site, respectively. As a result, eight sets of concentration gradients of bovine insulin were obtained, ranging from 6.77 to 1.36 mg/mL, with a decrement of 0.77 mg/mL (50 mM HCl, 75 mM NaCl, and 20 µM ThT).

**Figure 3:**
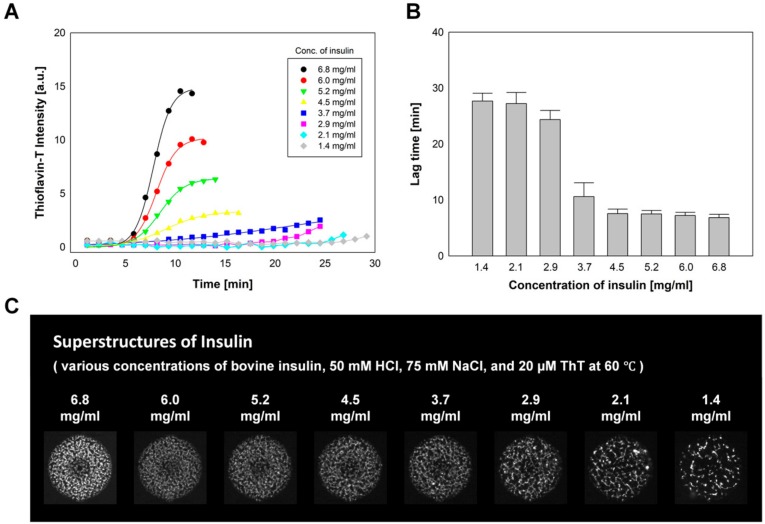
The effect of insulin concentration on insulin fibrillation. (**A**) The fluorescent intensity changes of ThT as a marker of insulin fibrillation. (**B**) Average lag times for insulin fibril formation (*n* = 8) and (**C**) the formation of insulin superstructures at various concentrations of insulin (50 mM HCl, 75 mM NaCl, and 20 µM ThT).

Figure 3A shows one set of the measured fibrillation rates of bovine insulin as a function of insulin concentration. The insulin fibrillations progressed through a lag phase where ThT fluorescence was not detected and a growth phase by increasing the ThT intensities until a final steady state. Average lag times for insulin fibril formation (*n* = 8) are shown in Figure 3B. The fastest insulin aggregation was observed at the highest insulin concentration, and the rate of insulin fibrillation decreased according to the decrease in insulin concentration. Figure 3C shows the formation of insulin fibrillar structures in incubation chambers after a 90 min incubation. Longer incubation times of up to 180 min did not result in further changes in fibrillar structure formation. Fluorescent imaging of eight incubation chambers containing various concentrations of insulin showed differences in the density of the protein fibrillar structure. As opposed to spherulites, which consist of radially oriented amyloid fibrils from an empty core [12,13,45], the fibrillar structures formed in the microfluidic chambers in this study exhibited a random orientation. The formation of dense fibril networks (or superstructures) was observed at high insulin concentrations by acquiring time-lapse fluorescence microscopy images of the incubation chambers. The initiation and growth of the superstructure were traced by acquiring time-series fluorescence microscope images of the incubation chambers.

### 2.4. The Effect of NaCl Concentration on Insulin Fibrillation

**Figure 4:**
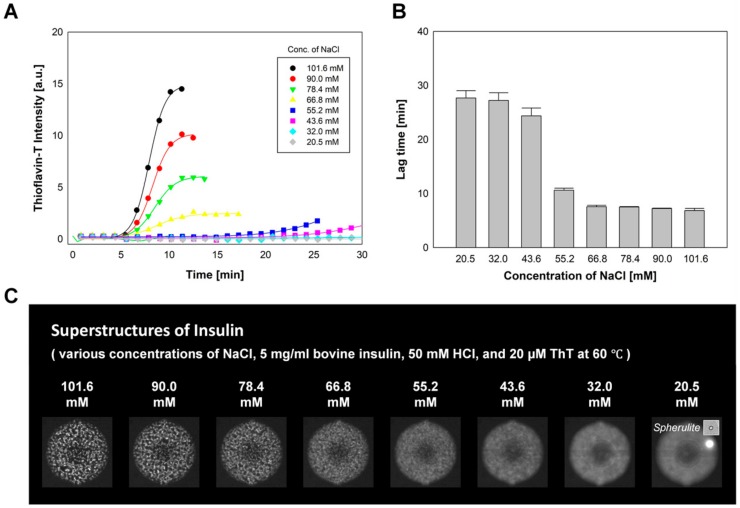
The effect of NaCl concentration on insulin fibrillation. (**A**) The rates of insulin fibrillation and (**B**) average lag times for insulin fibril formation (*n* = 8) at various NaCl concentrations (5 mg/mL bovine insulin, 50 mM HCl, and 20 µM ThT). (**C**) Fluorescent images of insulin superstructures formed in microfluidic incubation chambers. The inset shows the bright-field microscope image of a spherulite.

A significant increase in the rate of insulin fibrillation as a result of the addition of NaCl has been reported in insulin incubation experiments due to the ion–protein interactions during the aggregation process [5,7,46]. To investigate the effect of the NaCl concentration on insulin fibrillation in the microPAD, a concentration gradient of NaCl was created while the concentration of bovine insulin was kept constant. The NaCl concentration was varied from 101.6 to 20.5 mM, with a decrement of 11.6 mM (5 mg/mL bovine insulin, 50 mM HCl, and 20 µM ThT) by loading HCl solution (50 mM HCl and 20 µM ThT), NaCl solution (300 mM NaCl, 50 mM HCl and 20 µM ThT), HCl solution (50 mM HCl and 20 µM ThT), and bovine insulin solution (20 mg/mL bovine insulin, 40 mM HCl and 20 µM ThT) into the dilution solution-, factor #1-, factor #2-, and main factor-loading site, respectively. The final concentrations of insulin, HCl, and ThT were 5 mg/mL, 50 mM, and 20 µM, respectively, in all incubation chambers. Figure 4A exhibits one set of the fibrillation rates of bovine insulin at different NaCl concentrations. An increase in the rate of bovine insulin fibrillation as a result of increased NaCl concentration was observed in eight incubation chambers. Figure 4B shows average lag times for the formation of insulin fibrils (*n* = 8). Figure 4C shows the formation of insulin fibrillar structures at various concentrations of NaCl after a 90 min incubation. The highly crowded superstructures were observed in the case of incubations at high NaCl concentrations, and a decrease in NaCl concentration led to a decrease in the density of fibril superstructures. At the concentrations of NaCl below 43.6 mM, spherulites formed rather than random fibrillar (super)structures.

It is worth mentioning that the insulin fibrillation rate in our microfluidic device was higher than the fibrillation rate observed in conventional incubation [5,7,9] as well as in other microfluidic platforms [22]. The rapid on-chip insulin fibrillation was suggested to be mainly affected by the small reactor volume [22], but also the hydrophobicity of polydimethylsiloxane (PDMS) surface of the device expectedly increases the insulin fibrillation rate [1,7]. With the device developed here, we succeeded in reproducing eight sets of conventional incubation experiments in a single experiment. The two on-chip incubations, one with varying concentrations of insulin at a constant concentration of NaCl and the other with different concentrations of NaCl at a fixed concentration of insulin, shows explicit agreement on the effect of the two parameters on insulin fibrillation. Hence even the effect of an unknown parameter on insulin fibrillation can be evaluated by applying a new parameter with unknown effect, with one of the well-defined parameters for on-chip insulin incubations.

### 2.5. The Combined Effects of Different Concentrations of NaCl and HCl on Insulin Fibrillation

**Figure 5:**
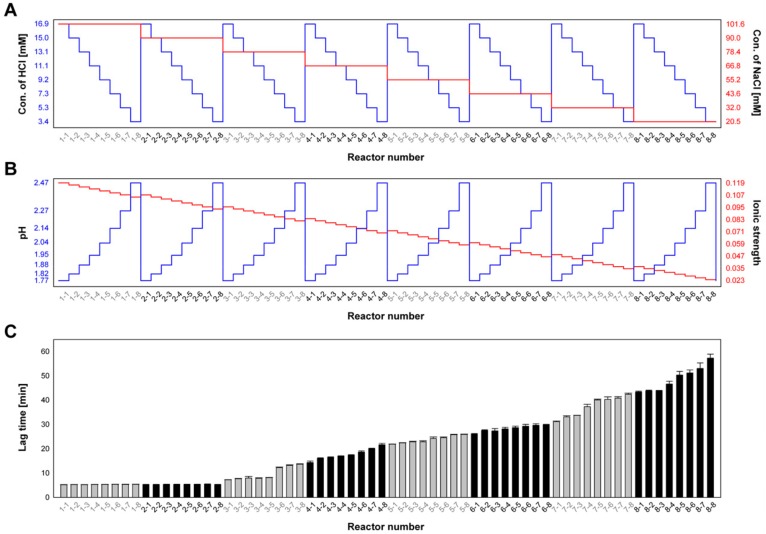
The combined effect of concentrations of NaCl and HCl on insulin fibrillation. (**A**) Concentrations of HCl (blue) and NaCl (red), (**B**) calculated pH (blue) and ionic strength (red) values, and (**C**) measured lag times for the formation of insulin fibril (*n* = 3) in the 64 incubation chambers.

To evaluate the combined effects of NaCl and HCl concentrations on insulin fibrillation, dual concentration gradients of NaCl and HCl were formed in the microPAD. Milli-Q water, HCl solution (50 mM HCl and 20 µM ThT), NaCl solution (300 mM NaCl and 20 µM ThT), and bovine insulin solution (20 mg/mL bovine insulin, 50 mM HCl, and 20 µM ThT) were introduced into the dilution solution-, factor #1-, factor #2-, and main factor-loading site, respectively. At a constant concentration of bovine insulin of 5 mg/mL, the concentration of NaCl ranged from 101.6 to 20.5 mM, with a decrement of 11.6 mM, and the concentration of HCl varied from 16.9 to 3.4 mM, with a decrement of 1.9 mM. Figure 5 shows HCl and NaCl concentrations (Figure 5A), calculated pH and ionic strength values (Figure 5B), and measured lag times for the formation of insulin fibrils (Figure 5C) in 64 chambers. The lag times decreased with an increase in the NaCl concentration, at a constant HCl concentration and with an increase in the HCl concentration at a constant NaCl concentration. At NaCl concentrations of 101.6 and 90.0 mM, increased concentrations of HCl led to shorter lag times; however, a definite trend of lag time decreasing with increasing HCl concentration was observed in the concentration ranges of NaCl lower than 78.4 mM. The on-chip fibrillation experiments further showed that low pH and high ionic strength decreased the lag time of the insulin fibril formation, and that ionic strength is a dominant factor for insulin fibrillation when the ionic strength is higher than 0.09 mol/L.

**Figure 6:**
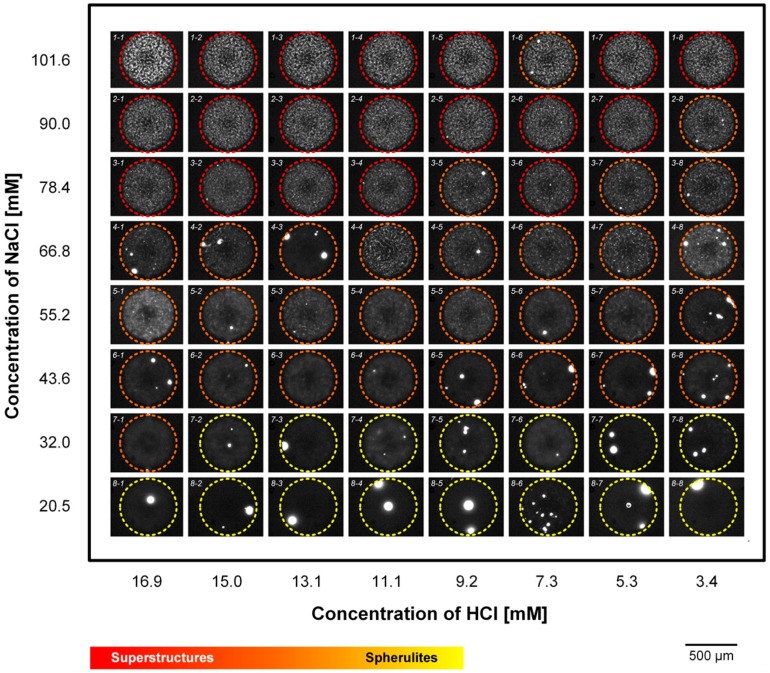
The combined effect of concentrations of NaCl and HCl on insulin fibrillar structure formation after 90 min of incubation.

Figure 6 shows the formation of fibril structures in 64 parallel chambers with various combinations of NaCl and HCl concentrations after a 90-min incubation. The red dashed circles indicate the formation of superstructures of bovine insulin and yellow dashed circles exhibit the formation of insulin fibrils. The intensity strength of the colors indicates the density of the formation of insulin fibrillar structures. At high concentrations of NaCl and HCl, relatively thick superstructures of insulin were observed, and the process of insulin fibrillation seems to be finalized. At the intermediate concentrations of NaCl and HCl, the formation of hairy but crowded fibrillar networks was observed. The formation of spherulites was found at low concentrations of NaCl and HCl, and the fibril structure formation was likely still ongoing. The spherulites were formed near the intermediate concentration of NaCl and HCl and were rarely observed at high concentrations of NaCl and HCl.

## 3. Materials and Methods

### 3.1. Materials

Insulin from bovine pancreas was obtained from Sigma-Aldrich (Zwijndrecht, The Netherlands) and dissolved at 20 mg/mL protein concentration in deionized water from Milli-Q filtration system (Millipore Co.), along with 50 mM HCl (Sigma-Aldrich, Zwijndrecht, The Netherlands) and 20 µM ThT (Sigma-Aldrich, Zwijndrecht, The Netherlands). Dilution buffer solution (50 mM HCl and 20 µM ThT), NaCl solution (300 mM NaCl, 50 mM HCl, and 20 µM ThT), and neutral buffer solution (20 µM ThT, pH 7.0) were prepared with Milli-Q water. NaCl solution was filtered with a 0.2 µm syringe filter (Whatman PLC, Sigma-Aldrich, Zwijndrecht, The Netherlands), to remove any residual solids.

### 3.2. Chip Fabrication

The microPAD consists of a PDMS fluidic layer and a PDMS control layer, which were fabricated by using the previously reported multilayer soft lithography technique [32,33]. Details of the fabrication process are described in Protocol S1 in the Supplementary Materials.

### 3.3. Simulation of the Actuation of Mixing Valves

Characterization of the mixing valves was processed based on stationary finite element simulations, using COMSOL MultiPhysics 5.1 (COMSOL MultiPhysics, Stockholm, Sweden) with the physics packages “Solid mechanics (solid)” and “Moving Mesh (ale)”. The model was built with the standard CAD kernel of COMSOL and is a simplification of the real device, i.e., it does not include the microchannels. The incubation chamber was considered half of an ellipsoid and the pressure chamber a cylinder. All domains were made of PDMS (density: 970 kg m^−3^, Young’s modulus: 0.7 GPa, Poisson’s ratio: 0.49), and the pressure was applied via a boundary load. The COMSOL model for the simulation of mixing valve actuation is provided in the Supplementary Materials.

### 3.4. Temperature Control

An indium tin oxide (ITO) heater and a temperature controller were purchased from Cell MicroControls (Norfolk, VA, USA). The controller was calibrated to adjust and control the temperature in the fluidic channels of the device. Details about the temperature control setup are provided in Figure S3 in the Supplementary Materials.

### 3.5. PDMS Membrane Valve Operation

The fluid flow in the microfluidic devices was controlled with a pneumatic control system. Microvalves were operated by applying compressed nitrogen gas into control channels. The pneumatic control system was automated by combining precision pressure regulators, 3/2-way solenoid valves, and EasyPort USB digital I/O controller (all from Festo, Delft, The Netherlands). The pneumatic system was controlled by a custom-built LabVIEW program (National Instruments Co., Austin, USA).

### 3.6. Insulin Aggregation on a Chip

After loading reagents into incubation chambers, we mixed them by operating the mixing valves (0.1 Hz for 3 min), and the device was heated to 60 °C by controlling the ITO heater. Then, the monitoring of the aggregation processes in 64 chambers by ThT based fluorescence was initiated. ThT fluorescence is associated with the binding of the marker to protein fibrils [47,48].

### 3.7. Data Processing

An inverted fluorescent microscope (Olympus IX73, Olympus, Leiderdorp, The Netherlands) was used that was equipped with an automatic XY-stage (99S000, Ludl Electronic Products Ltd., NY, USA) and a digital camera (ORCA-ER, Hamamatsu Photonics Deutschland GmbH, Herrsching, Germany), for the acquisition of images, in order to monitor the microfluidic reactors. The stage and camera were interconnected by a custom-built LabVIEW program (National Instruments Co., Austin, USA), to automatically acquire images in predefined regions of interest with programmed time intervals. The fluorescent signal from ThT-bound insulin fibrils was acquired by a filter cube (excitation: 436 nm; emission: 480 nm, Chroma Technology Corp., Vermont, USA). The acquired images were processed and analyzed by the time-series analyzer of Image J software (http://rsb.info.nih.gov/ij/). For the kinetic study of fibril formation, the obtained ThT fluorescence intensities were plotted as a function of time and fitted by a sigmoidal curve by using SigmaPlot (Systat Software Inc., San Jose, USA). The lag times for the formation of fibrils under various incubation conditions were determined by Equation S1 in the Supplementary Materials.

## 4. Conclusions

In this work, we established a high-throughput method to study protein aggregation, using 64 parallel incubation chambers on a single microfluidic chip. We presented the creation of nonlinear concentration gradients of HCl and NaCl and investigated their influences on insulin aggregation. The kinetics of fibril formation and the morphology of fibrillar structures under different conditions were investigated. The microPAD device developed here may be a useful tool for rapid evaluation of amyloid growth and the formation of fibrillar structures associated with many misfolding-based diseases, such as Alzheimer’s and Parkinson’s disease.

## Figures and Tables

**Table 1:** Compositions and combinations of the reagents in the 64 microfluidic incubation chambers.

Reactor Number	Final Concentration			Reactor Number	Final Concentration		
	Main factor (I_M_: Initial Conc.)	Factor #1 (I_F1_: Initial Conc.)	Factor #2 (I_F2_: Initial Conc.)		Main Factor (I_M_: Initial Conc.)	Factor #1 (I_F1_: Initial Conc.)	Factor #2 (I_F2_: Initial Conc.)
1-1	0.25 I_M_	0.34 I_F1_	0.34 I_F2_	5-1	0.25 I_M_	0.34 I_F1_	0.18 I_F2_
1-2	0.25 I_M_	0.30 I_F1_	0.34 I_F2_	5-2	0.25 I_M_	0.30 I_F1_	0.18 I_F2_
1-3	0.25 I_M_	0.26 I_F1_	0.34 I_F2_	5-3	0.25 I_M_	0.26 I_F1_	0.18 I_F2_
1-4	0.25 I_M_	0.22 I_F1_	0.34 I_F2_	5-4	0.25 I_M_	0.22 I_F1_	0.18 I_F2_
1-5	0.25 I_M_	0.18 I_F1_	0.34 I_F2_	5-5	0.25 I_M_	0.18 I_F1_	0.18 I_F2_
1-6	0.25 I_M_	0.15 I_F1_	0.34 I_F2_	5-6	0.25 I_M_	0.15 I_F1_	0.18 I_F2_
1-7	0.25 I_M_	0.11 I_F1_	0.34 I_F2_	5-7	0.25 I_M_	0.11 I_F1_	0.18 I_F2_
1-8	0.25 I_M_	0.07 I_F1_	0.34 I_F2_	5-8	0.25 I_M_	0.07 I_F1_	0.18 I_F2_
2-1	0.25 I_M_	0.34 I_F1_	0.30 I_F2_	6-1	0.25 I_M_	0.34 I_F1_	0.15 I_F2_
2-2	0.25 I_M_	0.30 I_F1_	0.30 I_F2_	6-2	0.25 I_M_	0.30 I_F1_	0.15 I_F2_
2-3	0.25 I_M_	0.26 I_F1_	0.30 I_F2_	6-3	0.25 I_M_	0.26 I_F1_	0.15 I_F2_
2-4	0.25 I_M_	0.22 I_F1_	0.30 I_F2_	6-4	0.25 I_M_	0.22 I_F1_	0.15 I_F2_
2-5	0.25 I_M_	0.18 I_F1_	0.30 I_F2_	6-5	0.25 I_M_	0.18 I_F1_	0.15 I_F2_
2-6	0.25 I_M_	0.15 I_F1_	0.30 I_F2_	6-6	0.25 I_M_	0.15 I_F1_	0.15 I_F2_
2-7	0.25 I_M_	0.11 I_F1_	0.30 I_F2_	6-7	0.25 I_M_	0.11 I_F1_	0.15 I_F2_
2-8	0.25 I_M_	0.07 I_F1_	0.30 I_F2_	6-8	0.25 I_M_	0.07 I_F1_	0.15 I_F2_
3-1	0.25 I_M_	0.34 I_F1_	0.26 I_F2_	7-1	0.25 I_M_	0.34 I_F1_	0.11 I_F2_
3-2	0.25 I_M_	0.30 I_F1_	0.26 I_F2_	7-2	0.25 I_M_	0.30 I_F1_	0.11 I_F2_
3-3	0.25 I_M_	0.26 I_F1_	0.26 I_F2_	7-3	0.25 I_M_	0.26 I_F1_	0.11 I_F2_
3-4	0.25 I_M_	0.22 I_F1_	0.26 I_F2_	7-4	0.25 I_M_	0.22 I_F1_	0.11 I_F2_
3-5	0.25 I_M_	0.18 I_F1_	0.26 I_F2_	7-5	0.25 I_M_	0.18 I_F1_	0.11 I_F2_
3-6	0.25 I_M_	0.15 I_F1_	0.26 I_F2_	7-6	0.25 I_M_	0.15 I_F1_	0.11 I_F2_
3-7	0.25 I_M_	0.11 I_F1_	0.26 I_F2_	7-7	0.25 I_M_	0.11 I_F1_	0.11 I_F2_
3-8	0.25 I_M_	0.07 I_F1_	0.26 I_F2_	7-8	0.25 I_M_	0.07 I_F1_	0.11 I_F2_
4-1	0.25 I_M_	0.34 I_F1_	0.22 I_F2_	8-1	0.25 I_M_	0.34 I_F1_	0.07 I_F2_
4-2	0.25 I_M_	0.30 I_F1_	0.22 I_F2_	8-2	0.25 I_M_	0.30 I_F1_	0.07 I_F2_
4-3	0.25 I_M_	0.26 I_F1_	0.22 I_F2_	8-3	0.25 I_M_	0.26 I_F1_	0.07 I_F2_
4-4	0.25 I_M_	0.22 I_F1_	0.22 I_F2_	8-4	0.25 I_M_	0.22 I_F1_	0.07 I_F2_
4-5	0.25 I_M_	0.18 I_F1_	0.22 I_F2_	8-5	0.25 I_M_	0.18 I_F1_	0.07 I_F2_
4-6	0.25 I_M_	0.15 I_F1_	0.22 I_F2_	8-6	0.25 I_M_	0.15 I_F1_	0.07 I_F2_
4-7	0.25 I_M_	0.11 I_F1_	0.22 I_F2_	8-7	0.25 I_M_	0.11 I_F1_	0.07 I_F2_
4-8	0.25 I_M_	0.07 I_F1_	0.22 I_F2_	8-8	0.25 I_M_	0.07 I_F1_	0.07 I_F2_

## Supplementary Materials

The following are available online. Figure S1: Actuation of a mixing valve at various applied pressures. Figure S2: Mixing efficiency test. Protocol S1: Fabrication process of microfluidic devices. Figure S3: Temperature control setup. Equation S1: Kinetics of insulin fibril formation. Figure S4: Microfluidic device design for the optimization of operations. Movie S1: Device operation. Movie S2: Mixing valve operation, and COMSOL model file—the simulation of mixing valve actuation.
