# Supplementary material for: Systematic Investigation of Insulin Fibrillation on a Chip
Source: Molecules. 2020 Mar 18;25(6):1380. doi: 10.3390/molecules25061380 (PMC7144930; doi:10.3390/molecules25061380)

Supplementary Materials

Systematic Investigation of Insulin Fibrillation on a Chip

Hoon Suk Rho[1,2], Henk-Willem Veltkamp[3], Alexander T. Hanke[4], Marcel Ottens[4], Christian Breukers[5], Pamela Habibović[1], and Han Gardeniers[2,*]

[1]Department of Instructive Biomaterials Engineering, MERLN Institute for Technology-Inspired Regenerative Medicine, Maastricht University, The Netherlands
[2]Mesoscale Chemical Systems Group, MESA+ Institute for Nanotechnology, University of Twente, The Netherlands
[3]Integrated Devices and Systems Group, MESA+ Institute for Nanotechnology, University of Twente, The Netherlands
[4]BioProcess Engineering Group, Department of Biotechnology, Faculty of Applied Sciences, Delft University of Technology, The Netherlands
[5]Medical Cell BioPhysics Group, Technical Medical Centre, University of Twente, The Netherlands

Corresponding Author
* Mesoscale Chemical Systems
Faculty of Science and Technology
University of Twente
7500 AE Enschede
The Netherlands
Telephone # +31-(0)53-489-4356
j.g.e.gardeniers@utwente.nl

Contents

Figure S1. Actuation of a mixing valve at various applied pressures .... Page S2

Figure S2. Mixing efficiency test... Page S3

Protocol S1. Fabrication processes of microfluidic devices .... Page S4

Figure S3. Temperature control setup .... Page S5

Equation S1. Kinetics of insulin fibril formation .... Page S6

Figure S4. Design of a microfluidic device for the optimization of operations .... Page S7

Figure S1. Actuation of a mixing valve at various pressures. A. COMSOL MultiPhysics 5.1 (COMSOL MultiPhysics, Stockholm, Sweden) simulation of the actuation of the mixing valve at various applied pressures. B. Fluorescence microscopy images of the actuated mixing valve. The incubation chamber was filled with 100 μM of resorufin (Sigma-Aldrich, Zwijndrecht, The Netherlands) for the visualization of the displacement of the valve. The fluorescence signal from resorufin was monitored by a fluorescence filter (excitation: BP 515 - 560 nm; emission: LP 590 nm) to obtain the actuation height of the valve at different applied pressures [1]. C. Measurements of the actuation of the mixing valve at various applied pressures.

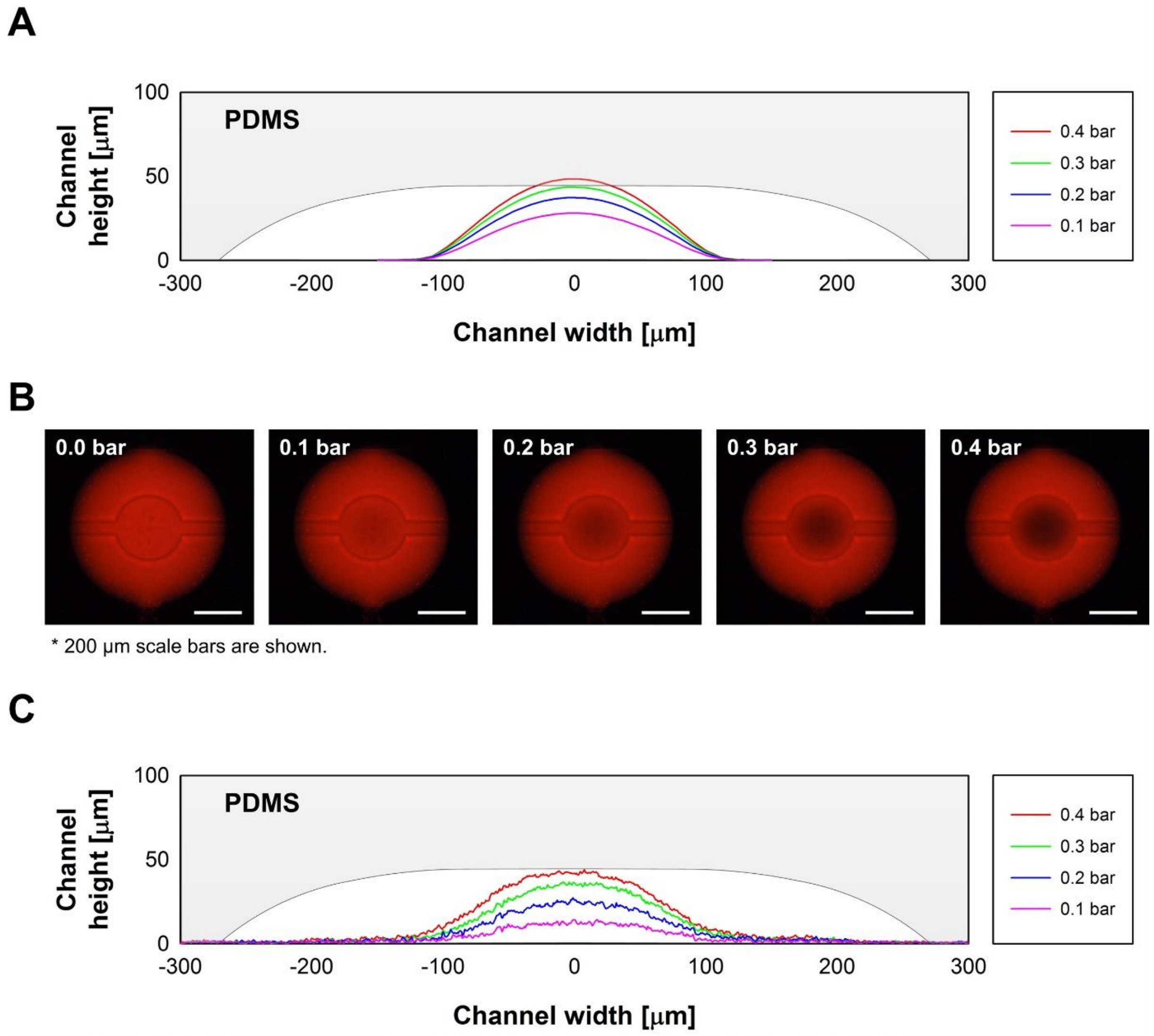

[1] Rho HS, Yang Y, Hanke AT, Ottens M, Terstappen LW, Gardeniers H. Programmable v-type valve for cell and particle manipulation in microfluidic devices. *Lab on a Chip*. 2016, 305-311.

Figure S2. Mixing efficiency test. A. Dilution solution-, factor #1, factor #2-, and main factor sites in a metering unit were filled with yellow, blue, red, green color dye solutions, respectively (a). Then, the dye solutions were introduced into incubation chambers (b) and mixed by operating mixing valves sequentially at 0.1 Hz (c, d). B. The mixing of the solutions was recorded, and the average brightness values of the incubation chamber areas were measured using Image J software. The average brightness values in the chambers increased corresponding to the progress of mixing and reached constant values when the mixing was completed.

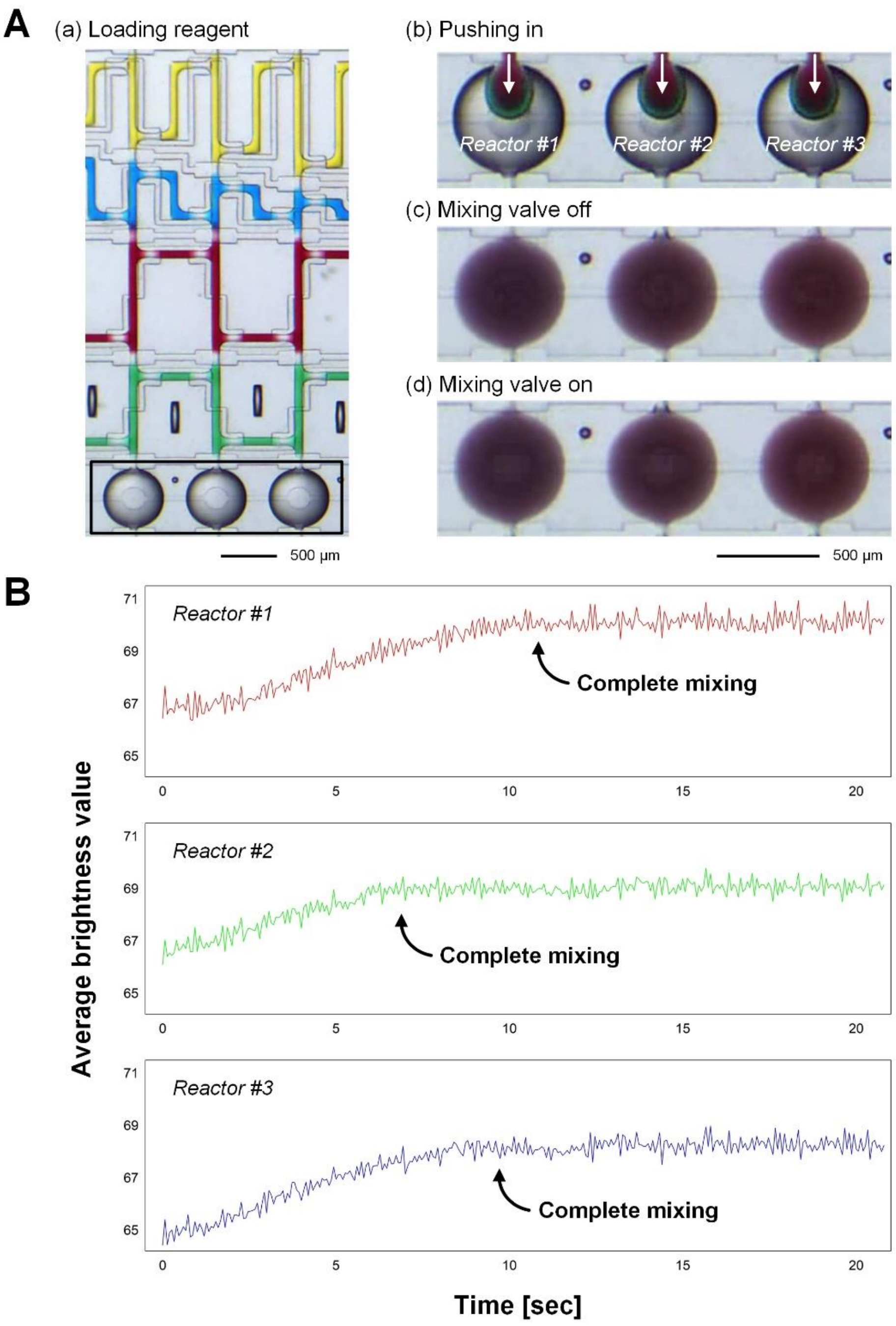

Protocol S1. Fabrication process of microfluidic devices.

Two photolithographic masks, one for a fluidic layer and the other for a control layer, were designed by CleWin software (WieWeb software, Hengelo, The Netherlands) and printed on 5” soda-lime glasses by LBPG Heidelberg DWL200 (Heidelberg Instruments Mikrotechnik GmbH, Germany). Positive photoresist (AZ 40XT-11D, MicroChemicals GmbH, Ulm, Germany) was spin-coated onto 4" silicon wafers. The wafers were exposed to UV light through masks and developed to achieve microstructures. The master mold for the fluidic layer was reflowed at 140 °C for one minute to obtain rounded channel profiles. The fluidic layer was made by pouring uncured PDMS (GE RTV615, prepolymer:curing agent = 7:1) onto the mold and curing for 45 minutes at 80 °C. Then, the fluidic layer was peeled off from the mold and holes for inlets and outlets were punched with a 25-gauge punch (Syneo Co., Angleton, USA). The control layer was produced by spinning uncured PDMS (prepolymer:curing agent = 20:1) onto the master mold at 2300 rpm for one minute and curing for 30 minutes at 80 °C. The fluidic layer was aligned over the control layer and the aligned layers were baked for 1 hour at 80 °C. The layers were peeled off from the mold and holes for the connections with control ports were punched using the same 25-gauge punch. The PDMS device was placed on a pre-cleaned glass slide (Fisher Scientific, Landsmeer, The Netherlands) and baked in the oven at 80 °C for 12 hours to enhance adhesion.

Figure S3. Temperature control setup. A commercial indium tin oxide (ITO) heater (Cell MicroControls, Norfolk, VA, USA) was used for elevating the temperature to induce protein aggregation. The optical transparency of the heater allowed microscopic monitoring of the protein folding process through the heater. To induce fibrillar structure formation in the fluidic channels with reagents the microfluidic device was placed onto the ITO heater (Figure S1A). A hole was punched in the fluidic layer of the device to connect a thermocouple for temperature readout. The controller calibrated the temperature continuously by applying a voltage to the ITO heater based on the readout. To validate the temperature control setup, bovine insulin aggregation was performed at two different temperatures, 50 °C and 60 °C. Previous studies showed a dramatic increase of the insulin fibrillation rate by increasing the temperature to 50 °C-60 °C [1, 2]. 10 mg/ml of bovine insulin (50 mM HCl, 75 mM NaCl, and 20 µM ThT) was loaded in a simple microfluidic device without metering units (the design is shown in Fig. S2) and the temperature wass increased up to 50 °C by the ITO heater. The change of ThT fluorescence intensity was used to monitor insulin fibrillation. The same experiment was repeated at 60 °C and the obtained ThT intensities were plotted against time in Figure S1B. The lag times for the formation of insulin fibrils at 50 °C and 60 °C were 136.1 ± 6.0 min and 0.7 ± 0.3 min, respectively. The apparent rate constants for the growth of fibrils at 50 °C and 60 °C were 0.1 ± 0.0 min^{-1} and 0.2 ± 0.0 min^{-1}.

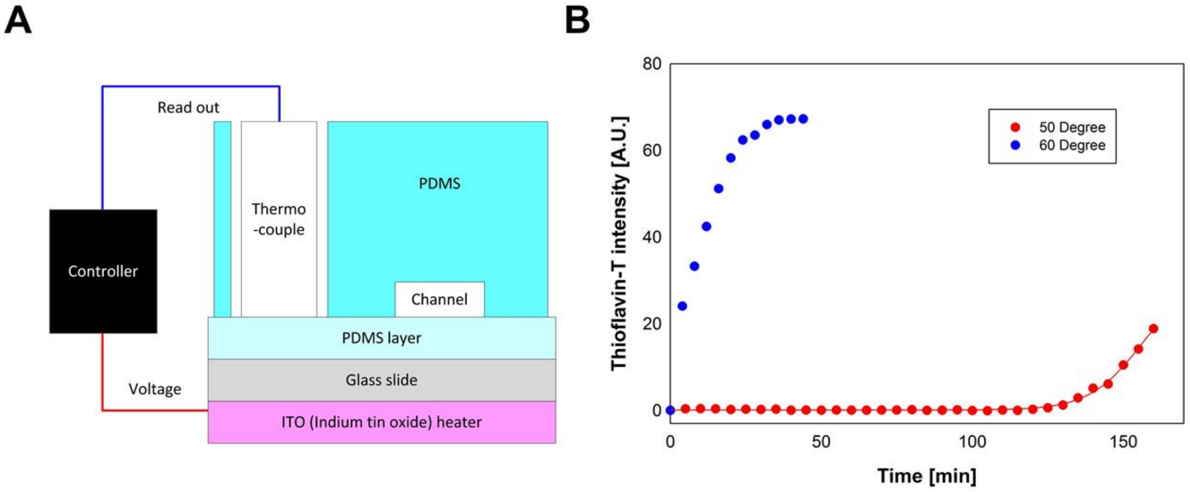

[1] Selkoe DJ. Folding proteins in fatal ways. *Nature*. 2003. 900-904.
[2] Brange J, Andersen L, Laursen ED, Meyn G, Rasmussen E. Toward understanding insulin fibrillation. *Journal of pharmaceutical sciences*. 1997. 517-525.

Equation S1. Kinetics of insulin fibril formation.

The obtained Thioflavin-T intensities were plotted as a function of time and fitted by a sigmoidal curve given by the equation

$$Y = y_i + m_i x + \frac{y_f + m_f x}{1 + e^{\frac{x - x_0}{a}}}$$

where

Y : Thioflavin-T intensity,

y_i : initial Thioflavin-T intensity,

y_f : final Thioflavin-T intensity, and

x_0 : 50% of maximum Thioflavin-T intensity [1].

Therefore,

Apparent rate constant $(k_{app}) = \frac{1}{a}$, and

Lag time $= x_0 - 2a$.

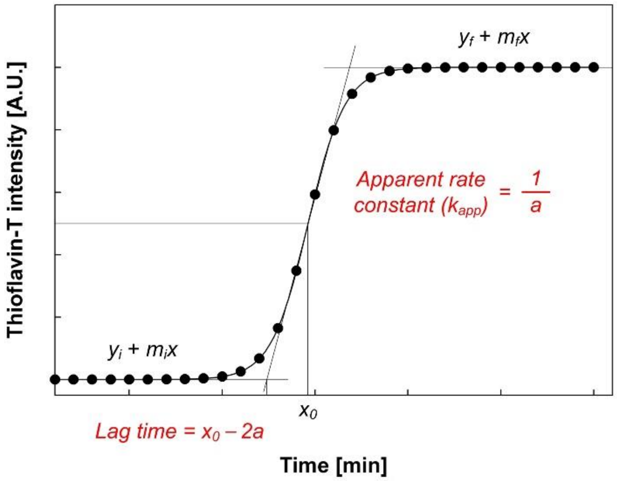

[1] Nielsen L, Frokjaer S, Brange J, Uversky VN, Fink AL. Probing the mechanism of insulin fibril formation with insulin mutants. *Biochemistry*. 2001. 8397-8409.

Figure S4. Microfluidic device design for the optimization of operations. The device was used for the incubation of 10 mg/ml of bovine insulin (50 mM HCl, 100mM NaCl, and 20 μM ThT) at different temperatures (50 °C and 60 °C) to calibrate a temperature control setup.

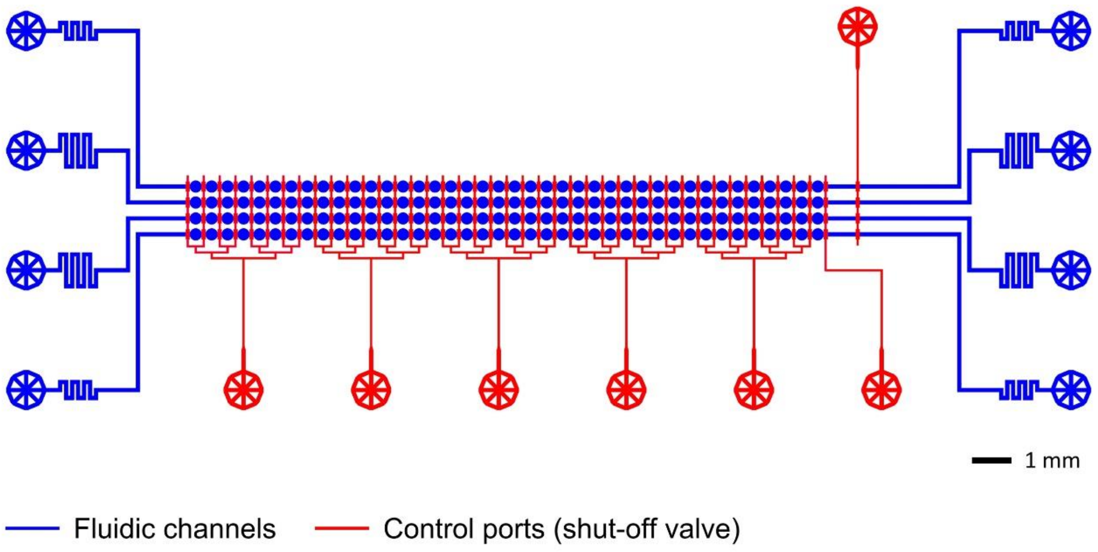

Supplement: Supplementary file 1 [file molecules-25-01380-s001.zip › New folder (2)/New folder.zip!/New folder/02 Supplementary_Insulin Fibrillation_Revised_V1.0.pdf]
